# Analysis of miRNA, mRNA, and TF interactions through network-based methods

**DOI:** 10.1186/s13637-015-0023-8

**Published:** 2015-06-04

**Authors:** Pietro H Guzzi, Maria Teresa Di Martino, Pierosandro Tagliaferri, Pierfrancesco Tassone, Mario Cannataro

**Affiliations:** 1 Department of Medical and Surgical Sciences, Magna Graecia University, Catanzaro, Italy; 2 Department of Experimental and Clinical Medicine, Magna Graecia University, Salvatore Venuta University Campus, Catanzaro, Italy; 3 Sbarro Institute for Cancer Research and Molecular Medicine, Center for Biotechnology, College of Science and Technology, Temple University, Philadelphia, PA USA

**Keywords:** mRNA, miRNA, Transcription factor, Network analysis, Data integration

## Abstract

Recent findings have elucidated that the regulation of messenger RNA (mRNA) levels is due to the synergistic and antagonist actions of transcription factors (TFs) and microRNAs (miRNAs). Mutual interactions among these molecules are easily modeled and analyzed using graphs whose nodes are molecules, and directed edges represent the associations among them. In particular, small subgraphs having three nodes also referred to as feed-forward loops (FFLs) or regulatory loops play a crucial role in many different diseases, such as cancer. Available technological platforms enable the investigation of only a single aspect of these mechanisms, e.g., the quantification of levels of mRNA or miRNA. Consequently, there exist different data sources for investigating some aspects of this problem, e.g., miRNA-mRNA or TF-mRNA associations. The comprehensive analysis is made possible only by the integration and the analysis of these data sources. Currently, the interest of researchers in this area is growing, the number of projects is increasing, and the number of challenges and issues for computer scientists is considerable. The need for an introductive survey from a computer science point of view consequently arises. This survey starts by discussing general concepts related to production of data. Then, main existing approaches of analysis are presented and discussed. Future improvements and challenges are also discussed.

## Review

### Introduction

The development of novel technological platforms in molecular biology has produced a large amount of data about different aspects of the *omic* world [1]. Consequently, the need for the development of novel approaches and methods to manage, store, and analyze this data arose [2–4]. In particular, this has caused the rise of a novel discipline, often referred to as *computational systems biology or network systems biology*, in which computer science, bioinformatics, and mathematical modeling play a synergistic role in the interpretation of large datasets belonging to different data sources [5, 6]. Network systems biology aims to discover basic principles of mutual interactions (or interplay) among different biological molecules (such as proteins, genes, or small fragments of non-coding nucleic acids) under the assumption that the information gathered from integrated analysis is higher than in the separate study of any data source [7, 8].

**Fig. 1:**
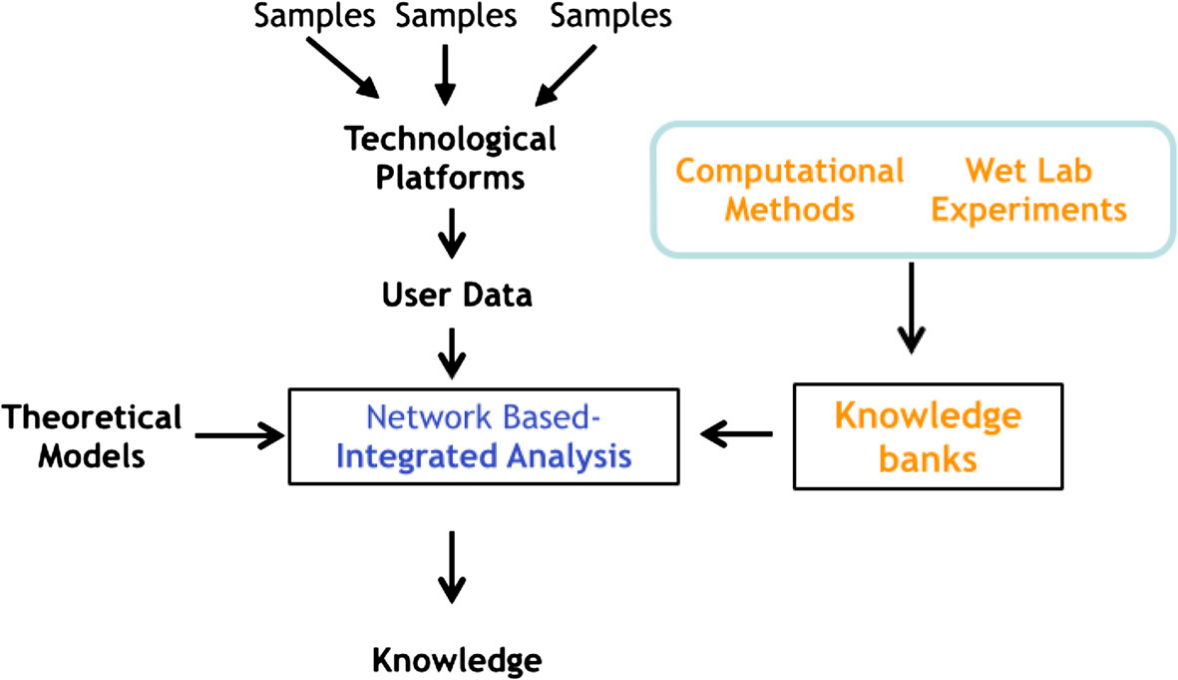
Flow of data. The figure depicts the flow of data in this field. The user may use different samples and different technological platforms to produce his/her own data. In parallel, previous wet lab experiments or computational experiments (e.g., prediction algorithms) have produced the availability of different knowledge banks. The network-based integrated analysis takes as input both user experimental data and knowledge banks and produces biologically meaningful knowledge using appropriate theoretical models

The flow of information in this field starts from technological platforms that produce different data about molecular biology as depicted in Fig. 1. Examples of such platforms are microarray for studying the expression of messenger RNA (mRNA) [9, 10] and microRNA (miRNA) [11], genomic microarrays for studying copy number variations (CNV) or single nucleotide polymorphisms (SNP), novel microarrays for studying non-coding RNAs (e.g., miRNA), genomic arrays for pharmacogenomics studies [12, 13], and novel next-generation sequencing (NGS) techniques. Classical approaches of analysis have produced a lot of information about the role of single class of molecules, but there is a lack of introduction of novel techniques aiming to analyze the interplay of molecules by integrating these data sources into a single comprehensive one [14, 15].

**Fig. 2:**
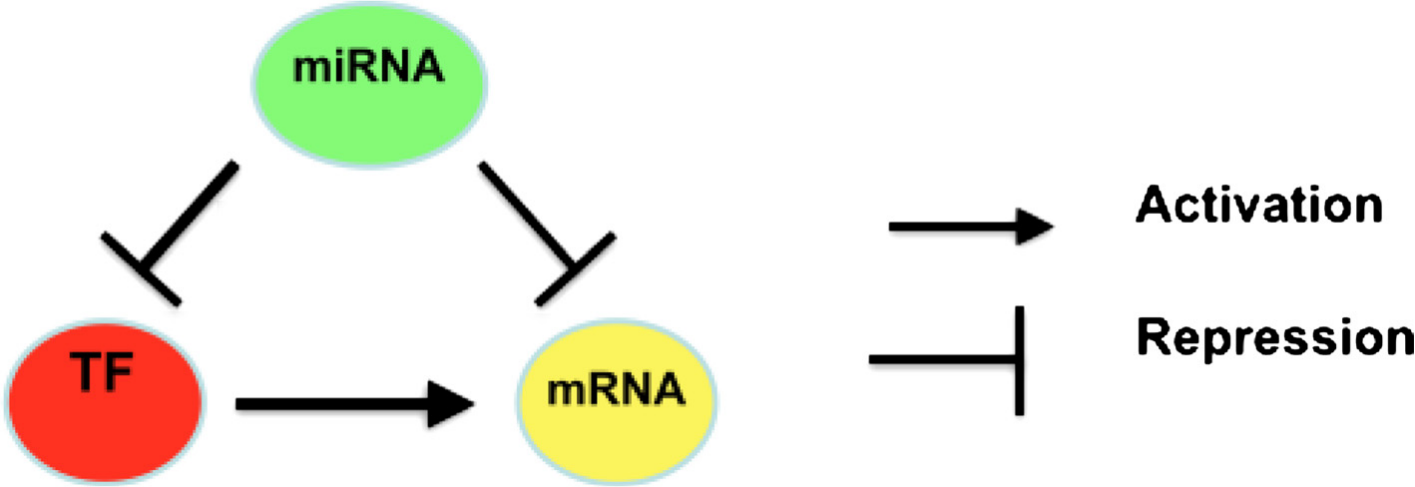
Example of a feed-forward loop. The figure depicts a simple graph modeling the interactions among miRNA (*green node*), mRNA (*yellow node*), and TF (*red node*) through two different kinds of edges. In particular, the figure depicts a miRNA that negatively regulates (repression) TF and mRNA and a TF that positively regulates (activation) a mRNA

Here we focus on the study of complex mechanisms of the regulation of gene expression. Recent results have confirmed that the transcription of mRNA into proteins is a multi-step process in which different molecules play a synergistic role [16]. In particular, miRNAs and transcription factors (TFs) play a direct role in the regulation of gene expression that results in variable levels of gene transcripts and proteins. Since there is not a direct technological platform to investigate these complex interactions, the integration of different datasets will become increasingly important as elucidated in the work by Muniategui et al. [17]. The integration of these datasets may be easily made by using models from graph theory. Consequently, it is possible to build comprehensive graphs in which nodes are miRNAs, mRNAs, and TFs, and directed edges connecting them represent the action of the molecules as depicted in Fig. 2. Edges are subdivided into (i) activation edges which represent a molecule whose action results in an increasing of the level of another one, and (ii) inhibition edges which connect a molecule whose action results in a decreasing of the levels of another one. Usually, edges connect a miRNA to a mRNA or a TF, or a TF to a gene [18]. Starting from this formalism, it is possible to extract small connected subgraphs with three different classes of nodes, representing feedback loops and feed-forward loops (FFLs) in which miRNAs participate together with transcription factors as depicted in Fig. 2.

The efforts of the scientific community have produced a set of projects regarding integrated data analysis based on graph theory. Because in recent years much work has been made in the study of TF and miRNA co-regulation, we think that there is a need to present in a systematic catalogue all the available methods. In this review, we summarize the types of regulatory networks. Future challenges and perspectives on TF-miRNA co-regulation are also discussed. Moreover, as a specific contribution of the presented work, we extended the work of [19] by discussing some recent approaches and by using a computer science perspective.

### Background

#### mRNA, miRNA, and transcription factor interactions

As stated in the central dogma of molecular biology, genes guide protein synthesis through mRNA molecules. Since the information contained in genes cannot be directly translated into proteins, information is at first transcribed into mRNA molecules. Each molecule of mRNA encodes the information for one protein. The mRNA molecules migrate through the nuclear envelope to the cytoplasm, where they are translated by the rRNA of ribosomes. Finally, each mRNA is translated into a polymer of amino acids: a protein. In an ideal case, the quantity of mRNA molecules should be directly related to the quantity of the related protein. In such a way, the investigation of the quantity of mRNA through microarray technology should enable the investigation of the quantity of produced proteins. Unfortunately, as suggested by experimental evidences, this process is made complex by the presence of regulatory mechanisms that directly influence the production of proteins. In particular, recent findings have elucidated the role of two main classes of molecules that influence positively and negatively the protein synthesis: miRNA and TF [20].

miRNA refers to a set of small RNA molecules composed of 21–23 nucleotides that do not encode any protein but participate as regulators in protein formation [21]. Recent studies demonstrated that miRNAs play an essential role in carcinogenesis because the disgregation of their activity may cause the development of tumor invasion and migration [22]. miRNAs also act as a possible new target for molecular target therapy of various cancers [23, 24]. Thus, there is an increasing interest for miRNA studies in clinical applications such as in serological diagnosis and molecular-targeted therapeutics [25]. TFs are modular proteins that regulate gene transcription through binding to the promoter region of target genes by their DNA-binding domains. In such a way, TFs may increase the gene expression levels and the consequent level of produced proteins.

#### Interaction databases

The interaction databases used by the works here surveyed fall into three main classes:

- Databases storing associations among miRNA and genes, i.e., storing which genes are targeted by miRNAs
- Databases storing the associations among TF and genes, i.e., storing which genes are targeted by TFs
- Databases storing the associations among TF and miRNA, i.e., storing which TFs are targeted by miRNAs

All of these databases may store both confirmed associations, i.e., associations supported by experimental evidences, and predicted associations, i.e., associations that are predicted by computational methods. The current scenario presents some main characteristics: (i) the number of confirmed associations is in general less than that of predicted ones, (ii) the number of false positives (i.e., not real associations) is considerable, and (iii) the level of overlap among databases is low. Consequently, all the approaches consider different data sources and integrate them in order to enhance the quality of considered associations.

The association among miRNAs and their target genes, i.e., genes up- or downregulated, is currently an increasing research area. Currently, there exist different prediction softwares, i.e., softwares that can predict possible genes regulated by a miRNA through machine learning approaches, and different technological platforms that are able to confirm these results in wet lab experiment [26]. As a result of the joint effort (both in silico and wet lab experiments), several databases that store the association among miRNAs and mRNAs are now available. Examples of these databases are Microcosm [27], microrna.org [28], DIANA-microT [29], miRDB [30], PicTar [31], PITA [32], RNA22 [33], and TargetScan [34].

Similar to miRNAs, the complete enumeration of all the interactions among TF and genes is far to be complete. Thus, information stored into databases is quite incomplete. Main experiments used for discovering TF-gene relations are immunoprecipitations (ChIP) followed by sequencing (ChIP-seq) or by microarray hybridization (ChIP-chip) [35]. Both techniques enable a high-throughput discovery of relations, but usually, they also generate a large number of false positives [36]. In parallel to these techniques, we should recall main computational approaches for predicting TF and for retrieving resulting information from databases.

For instance, the TRANSFAC database [37] is one of the main resources of experimentally verified TF targets from publications or databases. Similarly, CHEA [38] stores ChIP-seq and ChIP-chip data related to TF targets generated by different projects. The availability of different data sources with different reliabilities causes the need of integration of several of these methods and data to obtain comprehensive and accurate TF targets [18].

The third main knowledge source used by the works discussed in this survey is represented by databases storing information related to the regulation of miRNAs by TFs. The number of TF-miRNA regulation databases is lower than the number of the other two kinds of databases, because this approach is the youngest area of research. Examples of databases are TransmiR [39], TransFac [40], TargetScan [34], and PicTar [31].

### Network-based approaches for integrated analysis

#### A general model for integrating miRNA, mRNA, and TF data

All the approaches here discussed present some main characteristics. They have an internal knowledge base of associations extracted from literature and databases. The knowledge base is a comprehensive graph of associations. Nodes of these graphs fall into three classes representing respectively miRNAs, mRNAs, and TFs. Edges fall into two classes: activation and inhibition edges. A directed activation/inhibition edge connects a molecule that increases/decreases the level of another one. Main differences among the approaches are represented by the association databases that are used. This internal knowledge base is used for guiding the analysis of experimental data. Usually, experimental data are both miRNA and mRNA expression data taken from a pool of samples extracted from patients in case-control or time series experiments. For each patient, both mRNA and miRNA data are produced. Consequently, those experiments produced two expression vectors from each mRNA *m*_*i*_ and each miRNA *m*_*ij*_. Then, the expression vectors are correlated using some relatedness measures, such as Pearson correlation *ρ*(*m*_*i*_,*m* *i*_*j*_) for each mRNA-miRNA pair.

Then, data of knowledge bases are used to build the association graph from experimental data. This association graph is then mined to find small graphs representing FFL. The rest of the section presents some main approaches currently available for academic users. We should note that the literature also reports an approach of integration available for Ingenuity Pathway Analysis software that we do not report here since it is not freely available [41].

#### dChip-GemiNi (Gene and miRNA Network-based Integration)

dChip-GemiNi (Gene and miRNA Network-based Integration) [42] is a web server freely available for academic users which is able to integrate and analyze paired miRNA-mRNA expression data. The server side is written using the R programming language. Users may also download the source code for running it in a local environment. The ability of dChip-GemiNi has been tested by using some paired miRNA-mRNA datasets of solid cancers (liver, kidney, prostate, lung, and germ cell), and results are discussed in [42].

The workflow of analysis that has been used to build dChip-GemiNi contains four steps:

1. Initially, publicly available databases (e.g., TargetScan [34] for miRNA-mRNA association and data from TRANSFAC [40] for TF binding sites) have been used to construct TF-miRNA-gene networks, i.e., networks in which nodes are miRNA, genes, and TF, and edges represent the *regulates* relationship among them (e.g., a miRNA is connected to the target genes and a TF is connected to the target genes).
2. Then, experimental data (i.e., gene and miRNA expression profiles) are collected from publicly databases (e.g., GEO [43]).
3. Resulting networks (obtained in steps 1 and 2) are mined to extract significant motifs referred to as FFL motifs, i.e., small connected graphs in which there exist three different nodes (TF, miRNA, and mRNA) (see Fig. 2 for an example of FFL motifs).
4. Data of step 1 are used to further validate the statistical relevance of results through an ad hoc defined network motif score (NMS). The NMS is a function of multiple scores, including TF and miRNA binding scores to their target sequences, differential expression *P* values of the FFL components between normal and cancer tissues, and TF and miRNA’s target enrichment in differentially expressed genes and miRNAs.

**Fig. 3:**
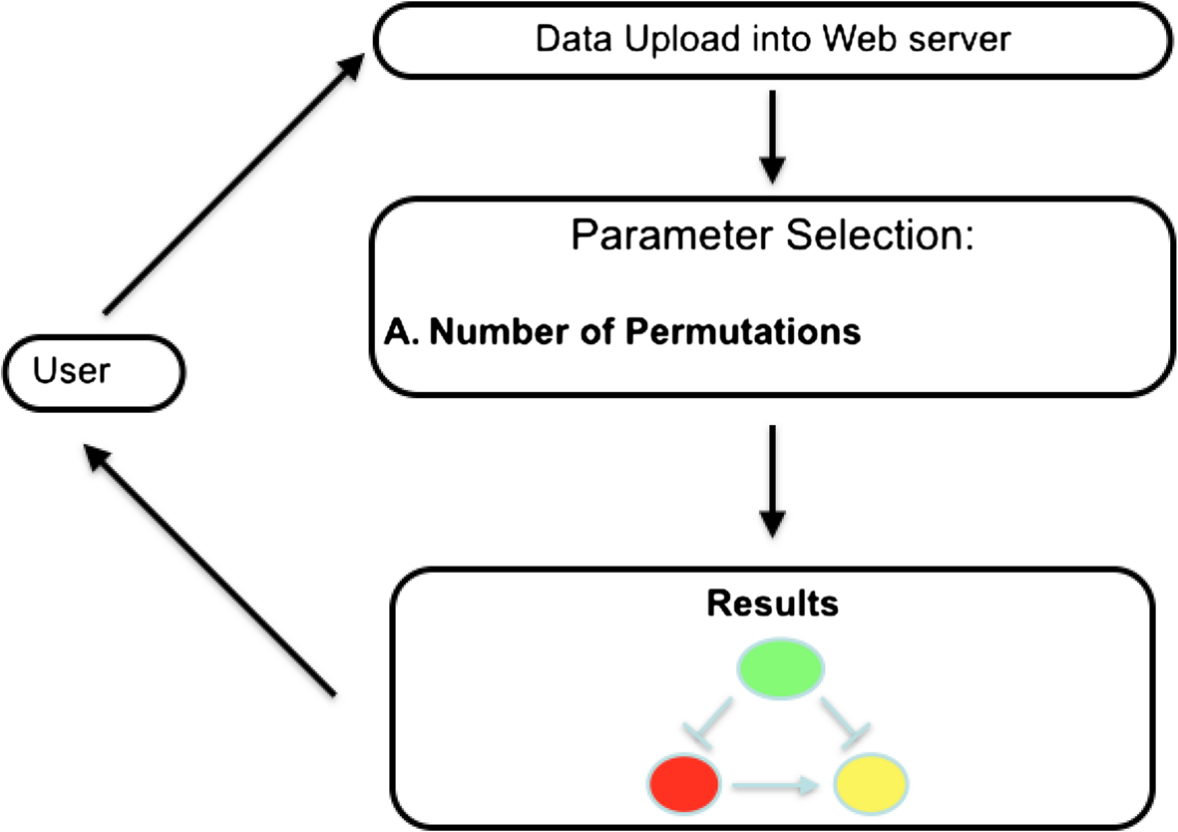
Workflow of analysis through dChip-GemiNi web server. The figure depicts the workflow of analysis of the dChip-GemiNi web server. Initially, the user has to upload datasets (both miRNA and mRNA) into the web server and to select the number of permutations (needed for the statistical evaluation of results). After the computation, results are presented to the user in a graphical way

As depicted in Fig. 3, when the user has to analyze experimental data, he/she has to start from two vectors of expression levels (one for mRNA and one for miRNA) obtained from experiments analyzing two conditions, e.g., normal and cancer. Data may be paired (i.e., for each sample, there exist both mRNA and miRNA) or non-paired (i.e., data belong to the same class but not to the same samples). Then, the user has to upload them into the web server and he/she receives as output a list of significant FFLs that are altered with respect to those used as the null model. dChip-GemiNi is also able to individuate FFLs consisting of TFs (i.e., genes that are able to regulate the expression of other genes), miRNAs, and their common target genes. In such a way, it can discover knowledge that cannot be discovered by the classical analysis. Experimental data are compared with respect to known associations among miRNAs, mRNAs, and TFs obtained from the literature and stored into the web server. TFs derived from literature are used as a null model to statistically rank predicted FFLs from the experimental data.

### MAGIA ^2^ web server

MAGIA ^2^ [44] is the evolution of the MAGIA web tool for the integrated analysis of both genes and microRNA. MAGIA ^2^ is deployed as a freely available web server. To build association networks, MAGIA ^2^ uses eight different databases of miRNA/mRNA associations: Microcosm [27], microrna.org [28], DIANA-microT [29], miRDB [30], PicTar [31], PITA [32], RNA22 [33], and TargetScan [34]. Such predictors are used to build the null models, i.e., associations that are known by literature. Regarding TFs, MAGIA ^2^ uses experimentally validated TF-miRNA interactions reported in mirGen2.0 [45] and TransmiR [39], whereas TF-gene interactions are obtained from ECRbase database [46].

The analysis through the MAGIA ^2^ web server starts by uploading data into the web server, usually a matrix for gene/transcripts and one for miRNA expression data. Data may belong to time series experiments in which for each sample there exists a pair miRNA/mRNA experiment (referred to as matched data), or a two-class experiment (referred to as un-matched data). Then, users have to select an association measure among mRNA and miRNA, i.e., a measure of relatedness among expression values. For matched experiments, MAGIA ^2^ offers the following measures: Pearson linear correlation, Spearman rank-based correlation, and an association measure based on information theory for time series experiments (referred to as matched). Diversely, for un-matched design, only a meta-analysis is possible.

The choice among measures is strictly dependent on the characteristics of data: for non-normally distributed data and/or small sample size experiments (e.g., 3–5), it is suggested to use Spearman correlation, which is a non-parametric rank-based linear measure, whereas for normally distributed data and medium-large sample size (more than 5 samples), authors suggest the use of the Pearson linear correlation measure; finally, for large sample size (more than 20 samples), it is suggested to use mutual information that is an information measure quantifying the mutual dependence of variables.

**Fig. 4:**
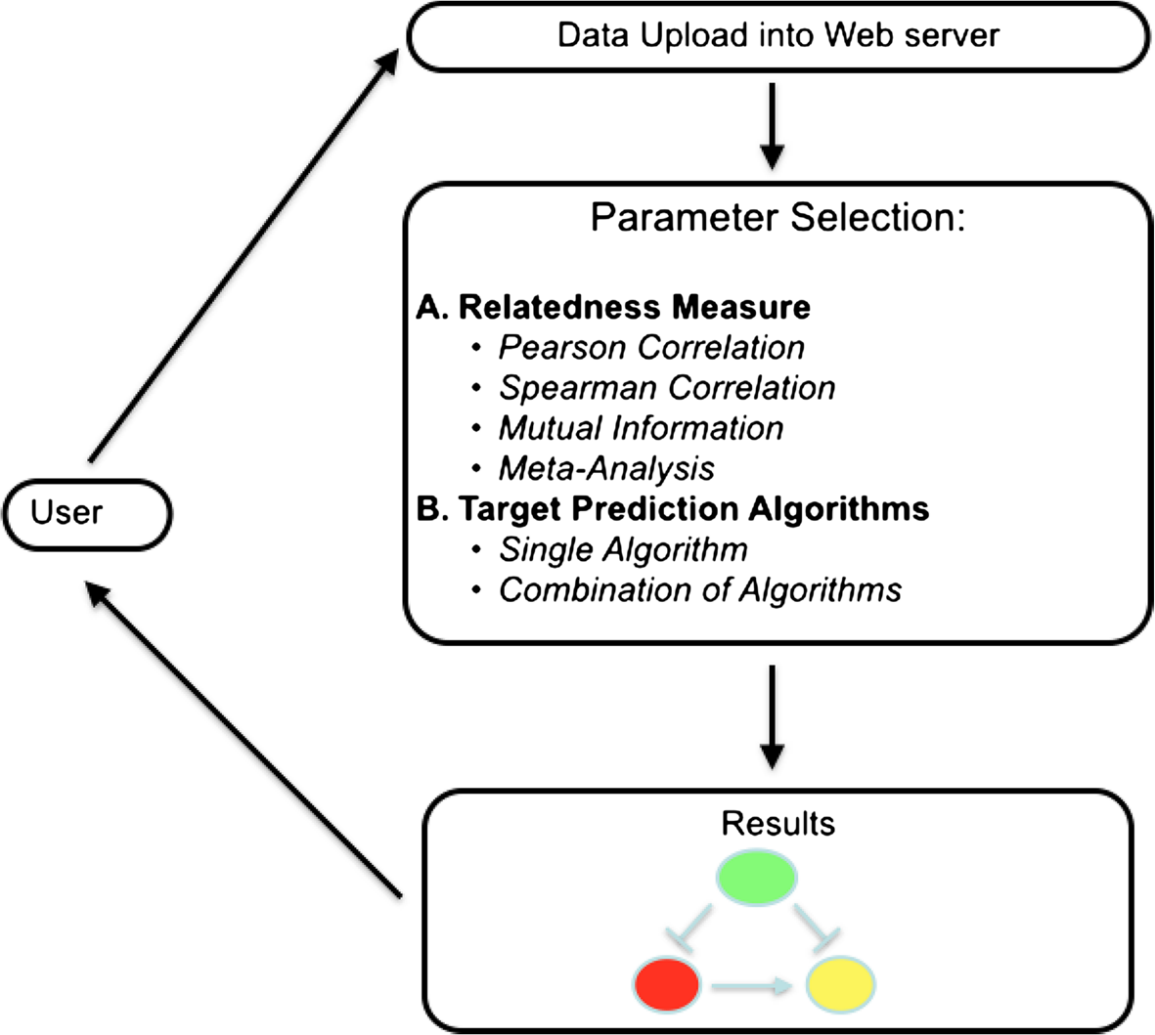
Workflow of analysis through MAGIA ^2^ web server. The figure depicts the workflow of analysis of the MAGIA ^2^ web server. Initially, the user has to upload datasets into the web server. Datasets must contain information of expression of mRNA and miRNA and may be paired (i.e., generated from time series experiments) or unpaired (i.e., generated from two-class experiments, e.g., healthy vs diseased). Then, the user has to select an appropriate measure of correlation among miRNA and mRNA (theoretical model) and the target prediction algorithms (knowledge banks). Finally, results are presented to the user in a graphical way

Diversely, for un-matched experiments, i.e., experiments in which samples are subdivided into two classes, the web server offers the meta-analysis approach that is based on the combination of *P* values of differential expression, separately for genes and miRNAs across sample classes. The user may also choose which databases are used to extract associations from those explained so far. In case of choice of multiple databases, search results may contain their union or intersection. Finally, experimentally derived associations are compared to those contained in the databases, and two kinds of networks are derived as depicted in Fig. 4.

#### mirConnX

**Fig. 5:**
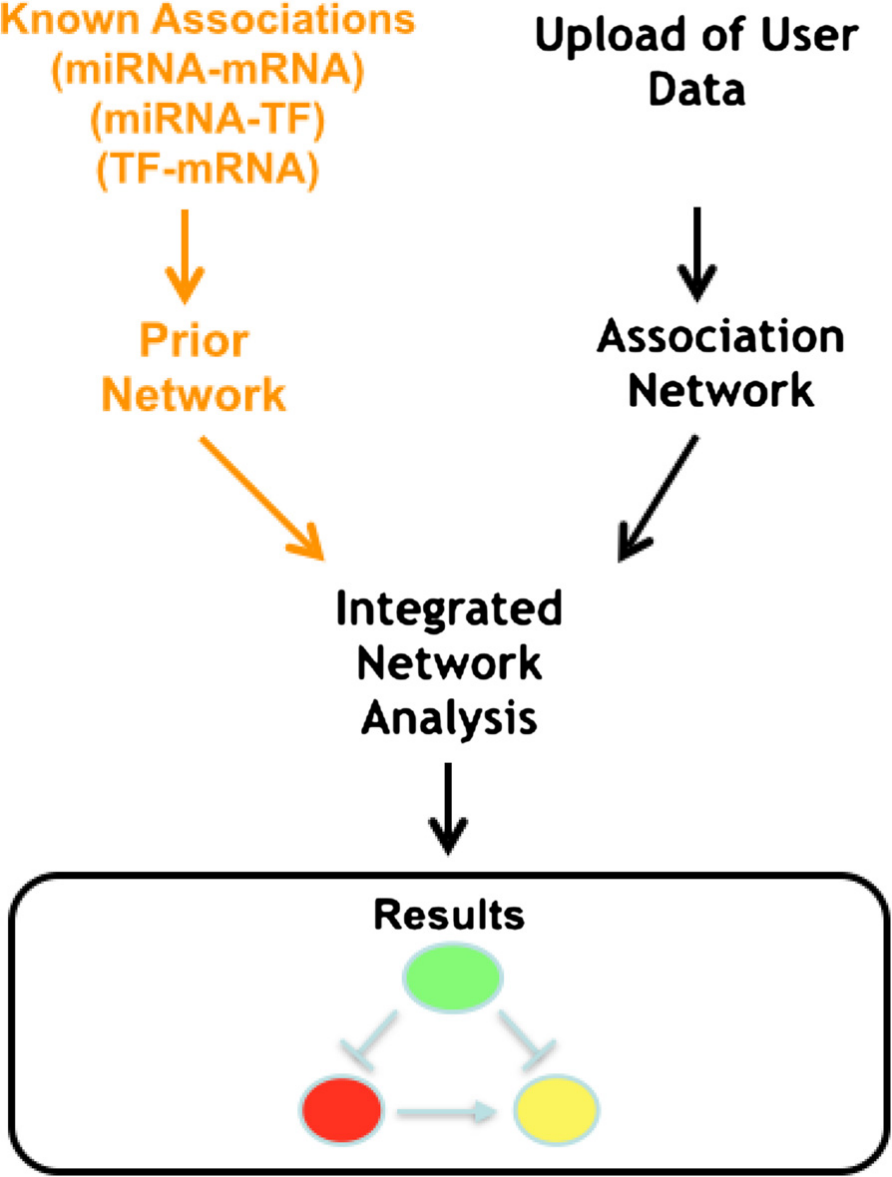
Analysis through mirConnX web server. The figure depicts the workflow of analysis on the mirConnX web server. Prior to the upload of user data, curators of the web server have produced a network of association among miRNAs, mRNAs, and TFs on a genome-wide scale for human and mouse. The user may upload his/her own data (miRNA and mRNA expression), and the web server builds the association network. Then, two networks are compared and FFLs are evidenced. Finally, results are presented to the user

mirConnX [47] is based on a broader perspective with respect to the previous approaches since it uses a genome-wide approach. Unfortunately, it enables only the analysis of data of two organisms: human and mouse. The workflow of analysis is based on the comparison of two networks of associations among genes, TFs, and mRNAs, as depicted in Fig. 5.

The first network, used as a null model, is derived from the analysis of databases and literature. In this network, nodes are miRNAs, TFs, and genes, and an edge connects two nodes when an association has been found. Examples of associations are (i) a miRNA that regulates a gene or a TF, or (ii) a TF that regulates a gene. Edges are weighted, and the weight reflects the strength of the association. miRNA targets are derived by integrating results stored in PITA [32], miRANDA [48], TargetScan 5.0 [34], RNAhybrid [49], Pictar [31], TarBase [50], and miRecords [51] databases. Similarly, associations among TF and genes are derived by integrating predictions stored in JASPAR [52] and TRANSFAC [37]. The integration step is based on a mathematical model which is able to derive a value of confidence for each prediction that is used as a weight for the resulting edge.

The network built from experimental data uploaded by the user is obtained by analyzing all the possible pairwise interactions between TFs, miRNAs, and genes across the samples/replicates. The user may choose different measures of associations, both parametric and non-parametric (e.g., Pearson, Spearman, and Kendall).

Finally, the software integrates the two networks via a simple weighted sum function (S) producing a novel network in which edges, which are found in both networks, have a greater weight. Results are finally visualized by using a Cytoscape-based interface [53] and all feed-forward loops, and their neighbors are evidenced. In addition, other simple analyses can be executed (e.g., an ontology-based analysis).

#### IntegraMiR

**Fig. 6:**
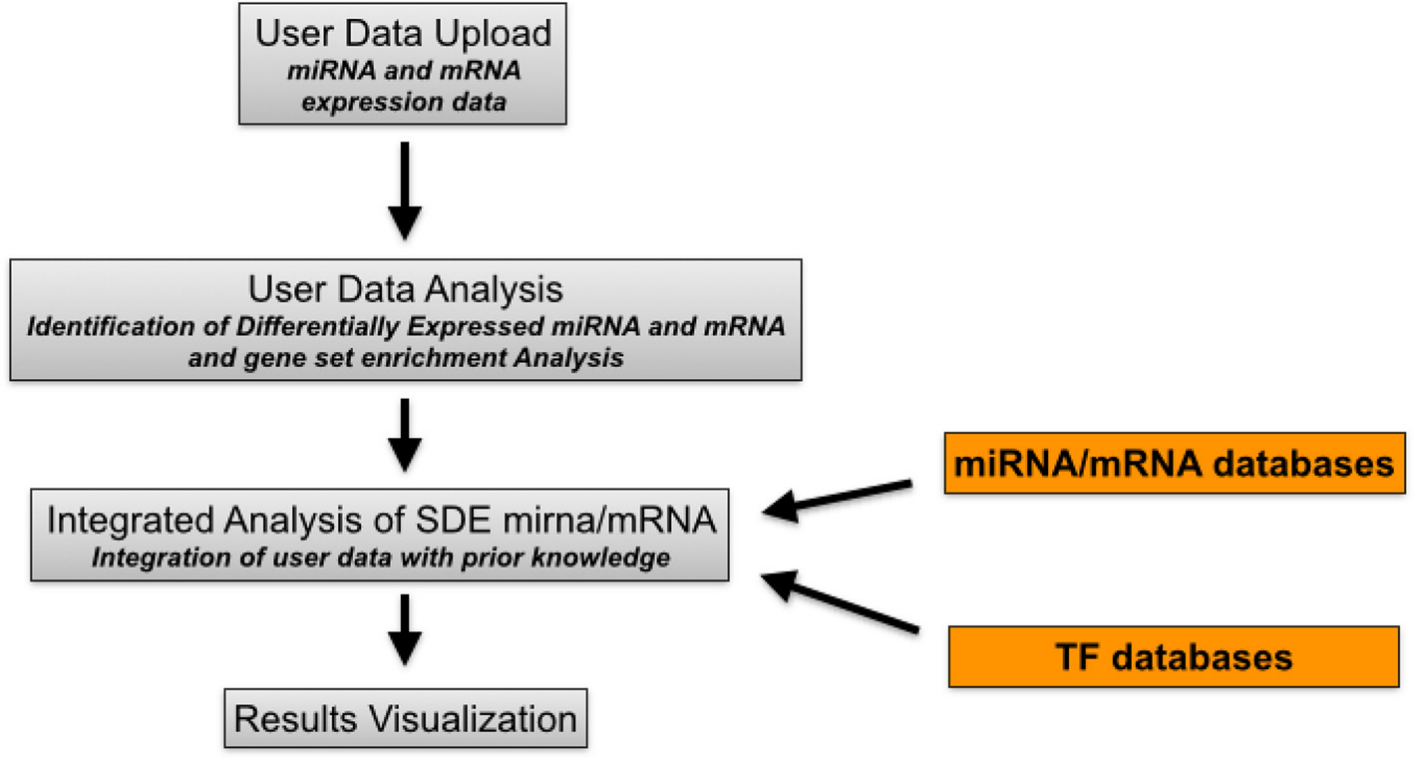
Workflow of analysis in IntegraMiR. IntegraMiR receives as input two lists of miRNA and mRNA expression data grouped into two classes (e.g., healthy vs diseased). Initially, the web server identifies significant differentially expressed (SDE) miRNAs and mRNAs. Then, prior knowledge is used to derive associations among miRNA, mRNA, and TF considering only those extracted in the previous steps. Then, FFLs whose members are differentially expressed are determined. These FFLs are then organized considering the kind of deregulation and ranked by using a statistical approach and visualized to the user

IntegraMiR [54] is a novel approach of integration of data that is based on the workflow depicted in Fig. 6. It receives as input mRNA and miRNA expression data, obtained from samples that are subdivided into two classes (e.g., controls vs. cases). It starts by searching for differentially expressed genes and mRNAs between two conditions by using the Bioconductor package LIMMA [55]. This step produces two lists, one for differentially expressed genes and one for differentially expressed miRNAs. Moreover, IntegraMiR uses LIMMA package to perform gene set enrichment analysis (GSEA), taking into account known biological knowledge about these transcripts to derive biological significance of both changed and unchanged transcripts. Then, associations among mRNA and miRNA are derived considering their individual expression levels (i.e., considering pairs of mRNA-miRNA whose regulation is inversely correlated) or through their target interactions—via functional analysis through literature and databases. Once this step is finished, IntegraMiR uses the TRANSFAC database [37] to derive associations among TFs and mRNAs and the TransmiR database [39] to derive associations among TFs and miRNAs. In particular, it focuses only on differentially expressed miRNA and mRNA. Thus, it can reconstruct FFLs whose members are differentially expressed. These FFLs are then organized considering the kind of deregulation and ranked by using a statistical approach and visualized to the user (see the original publication for a complete list of results). The software is available for download at (see the original publication for a complete list of results [54]).

#### Further analysis approaches

The current state of the art of research includes some other approaches of analysis that have been developed in different moments. Some of these approaches are not implemented in a single tool although they present a fully reproducible way to analyze miRNA-TF relationships [56].

For instance, Henriksen et al. [57] applied an integrated approach of analysis to identify miRNA-mRNA regulatory networks that are involved in glioma, a primary brain tumor. They identified miRNA functional targets during glioma malignant progression by combining the paired expression profiles of miRNAs and mRNAs of patients.

Nazarov et al. [58] developed an integrated analysis approach based on the use of different tools, both academic and commercials. The workflow of analysis is structured into different steps. They start from paired miRNA and mRNA data obtained from microarray experiments. In the first step, they pre-process miRNA and mRNA data using the Partek GS^®;^ platform in order to filter out non-relevant or out-of-quality data. Then, they use the LIMMA package of Bioconductor [59] to identify significant differentially expressed miRNA and mRNA. Then, they use Ingenuity Pathway Analysis (IPA)^®;^ to build regulatory networks of miRNA, mRNA, and transcription factors. In particular, they identify upstream regulators by using IPA. The IPA platform enables the reconstruction of causal networks constructed from individual relationships by providing a set of tools for inferring and scoring upstream regulators of gene expression data [41].

### Discussion

We here compare the so far discussed approaches by considering the following parameters:

- *Input and implementation*: We consider (i) the format of input (e.g., textual files or raw data), (ii) the experimental platforms (miRNA or mRNA), (iii) the design of the experiments (e.g., two-class experiments or time series), and (iv) the availability as a web server or as a stand-alone tool.
- *Analysis*: We consider the algorithmic approach (i.e., main characteristics of the analysis) and the main parameters customizable by the users.
- *Knowledge bases*: We consider which data sources have been used to derive associations among molecules, i.e., (i) miRNA-mRNA associations, (ii) TF-genes associations, and (iii) miRNA-TF associations.
- *Output*: We consider the characteristics of the output, its format (i.e., graphic or textual), as well as the possibility to link results to external knowledge bases (i.e., ontologies or semantic analysis [60]).

Considering Table 1, we should note at first that software available as web server (dChip-GemiNi, MAGIA ^2^, and mirConnX) are more user-friendly from a biological corner since the installation and running of R scripts is not easy without a bioinformatics support. Moreover, the MAGIA ^2^ web server enables the use of both two class and time series data, enhancing the possibility of analysis. All the softwares enable the use of different identifiers for genes, and some of them (e.g., dChip-GemiNi) have the possibility to use ad hoc identifiers. mirConnX has a main limitation on the input species since it may analyze only human and mouse data.

**Table 1:** Comparison of network-based analysis approaches considering availability and input data

Tool	Implementation	Input data	Input data grouping	Format of input data
dChip-GemiNi [42]	Web server - R script	mRNA-miRNA expression data grouped into two classes	Two-class data. Paired and not paired	Textual matrices
MAGIA ^2^[44]	Web server	mRNA-miRNA expression data. Time series and two-class data	Two-class and time series data	Textual matrices
mirConnX [47]	Web server	mRNA-miRNA expression data	Two-class data	Textual matrices. *Only human and mouse*
IntegraMiR [54]	R script	mRNA-miRNA expression data grouped into two classes	Two-class data	Textual matrices

Considering Table 2, we report that the MAGIA ^2^ web server is more flexible than the others since it gives to the user the possibility to choose different correlation measures and several target databases. Moreover, the user may intersect different databases. All the approaches compare experimental data with respect to knowledge bases, and in particular, mirConnX enables to weigh the influence of knowledge bases.

**Table 2:** Comparison of network-based analysis approaches considering algorithmic approach and parameters of analysis

Tool	Algorithmic approach	Parameters
dChip-GemiNi [42]	Identification of SDE genes. Building and analysis of experimental network	Permutations
MAGIA ^2^[44]	Building and analysis of experimental network	Relatedness measure. Target databases
mirConnX [47]	Comparison of networks	Association measure for experimental data. Weight of networks
IntegraMiR [54]	Building and analysis of experimental network	

Considering Table 3, we report that the MAGIA ^2^ web server used the largest number of association databases. In particular, we note that the most popular databases are TargetScan and Pictar (used by dChip-GemiNi, MAGIA ^2^, and mirConnX) for miRNA-mRNA associations and TRANSFAC for TF-gene association (used by dChip-GemiNi, mirConnX, and IntegraMiR).

**Table 3:** Comparison of network-based analysis approaches considering internal knowledge bases

Tool	miRNA-mRNA	TF-genes	miRNA-TF
dChip-GemiNi [42]	TargetScan and PicTar	TRANSFAC matrices v7.0	miRBase - TRANSFAC
MAGIA ^2^[44]	Microcosm, microrna.org, DIANA-microT, miRDB, PicTar, PITA, RNA22, and TargetScan	ECRbase	mirGen2.0 and TransmiR
mirConnX [47]	PITA, miRANDA, TargetScan 5.0, RNAhybrid, and PicTar	JASPAR and TRANSFAC	CoreBoost_HM
IntegraMiR [54]	mSigDB, mirTarBase, miRecords	TRANSFAC	TransMiR

Finally, considering the presentation of results, we note that the best performances are in generally achieved by using external visualizers (e.g., the Cytoscape web interface used by mirConnX or MAGIA ^2^). Moreover, mirConnX provides the possibility to link results to external databases (e.g., for enrichment analysis or search) (Table 4).

**Table 4:** Comparison of network-based analysis approaches considering output information

Tool	Kind	Format	Link to external knowledge bases
dChip-GemiNi [42]	Statistics of association. Visualization of network	Static	Not available
MAGIA ^2^[44]	Statistics of association. Visualization of network	Dynamic and exportable in Cytoscape	Available
mirConnX [47]	Statistics of association. Visualization of network	Dynamic	Available
IntegraMiR [54]	Lists of associated mRNA-miRNA and TF	Static	Not available

**Fig. 7:**
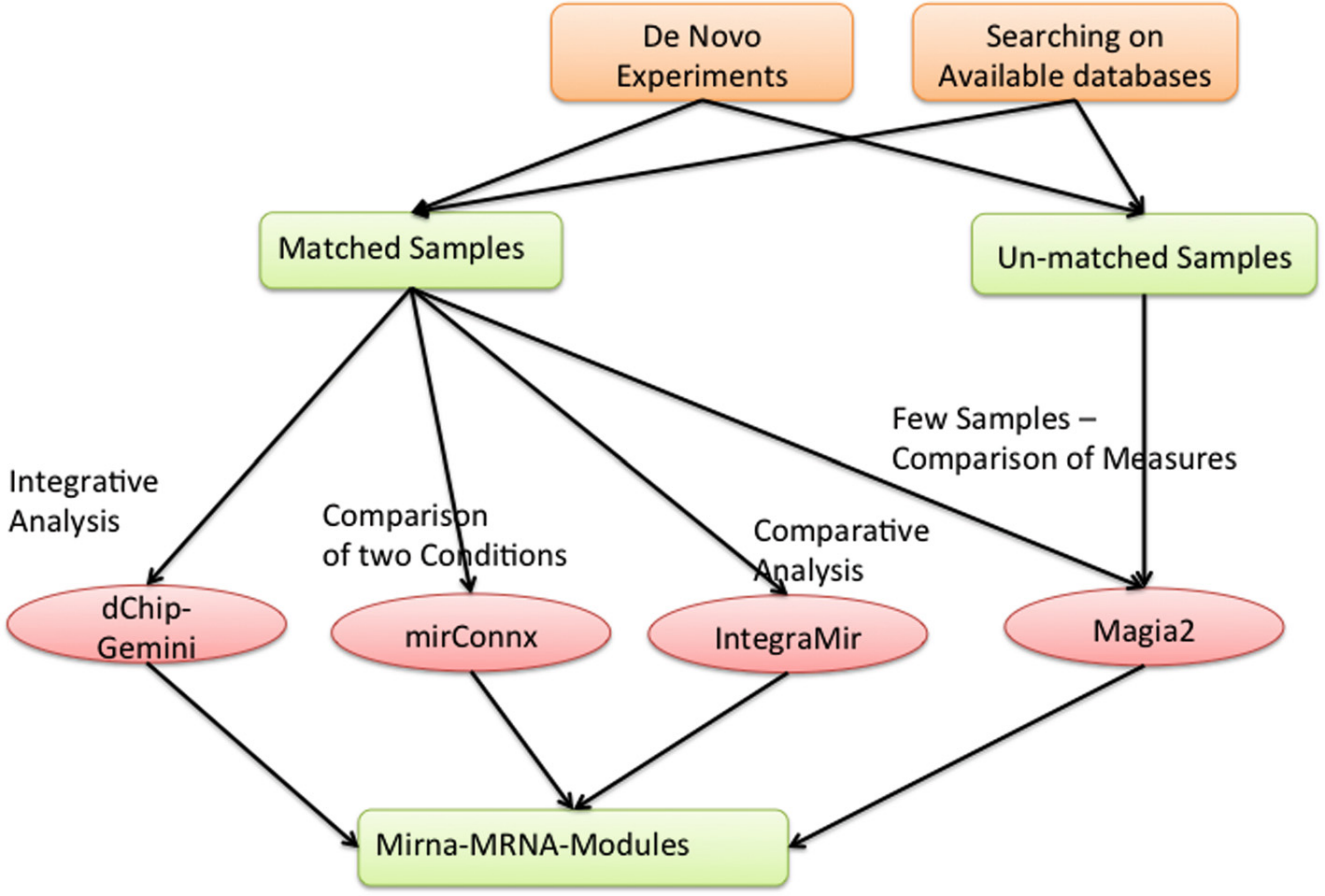
Integrated analysis of data. The figure shows typical workflow of integrated analysis of miRNA and mRNA data. The analysis starts by selecting samples. Users may choose to perform novel wet lab experiments or to download data from existing databases (e.g., Gene Expression Omnibus [61]). Data may belong to two main categories, e.g., matched and un-matched samples. In the first category, for each mRNA sample, there exists a corresponding miRNA sample and data are usually organized as time series. In the second category, data are grouped into classes. Currently, only the MAGIA ^2^ web server accepts as input both kinds of data. Considering the analysis of matched samples, the user may benefit from peculiarities of each software. For instance, MAGIA ^2^ offers the possibility to choose some functions suited for few samples. mirConnX enables the comparison of two conditions (e.g., healthy or diseases). IntegraMiR is particularly suited for expression data grouped into two classes (e.g., healthy vs diseased). dChip-Gemini is a general purpose software

Figure 7 reports some short examples of typical case studies by discussing main options and choices that are available to researchers.

## Conclusions

As evidenced before, the TF-miRNA-mRNA association represents undoubtedly a main resource for elucidating gene expression regulation at a systems level. The complete determination of miRNA and TF targets will enable a more powerful and reliable analysis. Consequently, from a technological point of view, the miRNA and TF target prediction and validation is still an urgent issue. In parallel, from a computational point of view, the integration of more data sources may improve the quality of analysis, since computational TF-miRNA regulatory networks are available for some genomes and diseases. Moreover, integrating TF-miRNA regulatory networks with other networks, such as functional networks (e.g., signaling pathways, metabolic pathways, protein-protein interaction networks) or semantic networks, will be an important improvement. This integration will aid in explaining how these networks regulate the biological processes and diseases at the systems level.
